# Drug-Induced Hypersensitivity Syndrome Accompanied With Acute Renal Failure With Hemodialysis: A Case Report and Literature Review

**DOI:** 10.7759/cureus.52335

**Published:** 2024-01-15

**Authors:** Ayako Setoyama, Yu Sawada

**Affiliations:** 1 Dermatology, University of Occupational and Environmental Health, Kitakyushu, JPN

**Keywords:** case report, literature review, allopurinol, acute renal failure, drug-induced hypersensitivity syndrome

## Abstract

Drug-induced hypersensitivity syndrome (DIHS) is a severe type of cutaneous adverse event involving systemic organ failures. In some cases of DIHS, acute renal failure takes place, and it becomes necessary to perform hemodialysis. However, the clinical outcome of renal failure in the course of treatment of DIHS remains unclear. Herein, we report a case of DIHS complicated with acute renal failure, which requires hemodialysis. Furthermore, we also review the DIHS cases accompanied by acute renal failure with hemodialysis in the English case report literature.

## Introduction

Drug-induced hypersensitivity syndrome (DIHS) is a severe type of cutaneous adverse event characterized by skin eruption, fever, and complications affecting blood and internal organs [1]. The representative culprits are usually anticonvulsants, antibiotics, and allopurinol [2]. The development of DIHS involves a multifaceted interaction among drugs, viruses, and the immune system, primarily orchestrated by T-cells [3-4]. DIHS commonly occurs two to six weeks after exposure to the causative drug [5]. In some cases of DIHS, acute renal failure takes place, and it becomes necessary to perform hemodialysis [6,7]. Among DIHS patients, 14.8% of cases experienced an increased serum creatinine, and 7.4% of cases required short-term supportive hemodialysis in DIHS patients [8]. However, the clinical outcome of renal failure in the course of treatment of DIHS remains unclear. Herein, we report a case of DIHS complicated with acute renal failure, which requires hemodialysis. Furthermore, we also review the DIHS cases accompanied by acute renal failure with hemodialysis in English case report literature.

## Case presentation

**Figure 1:**
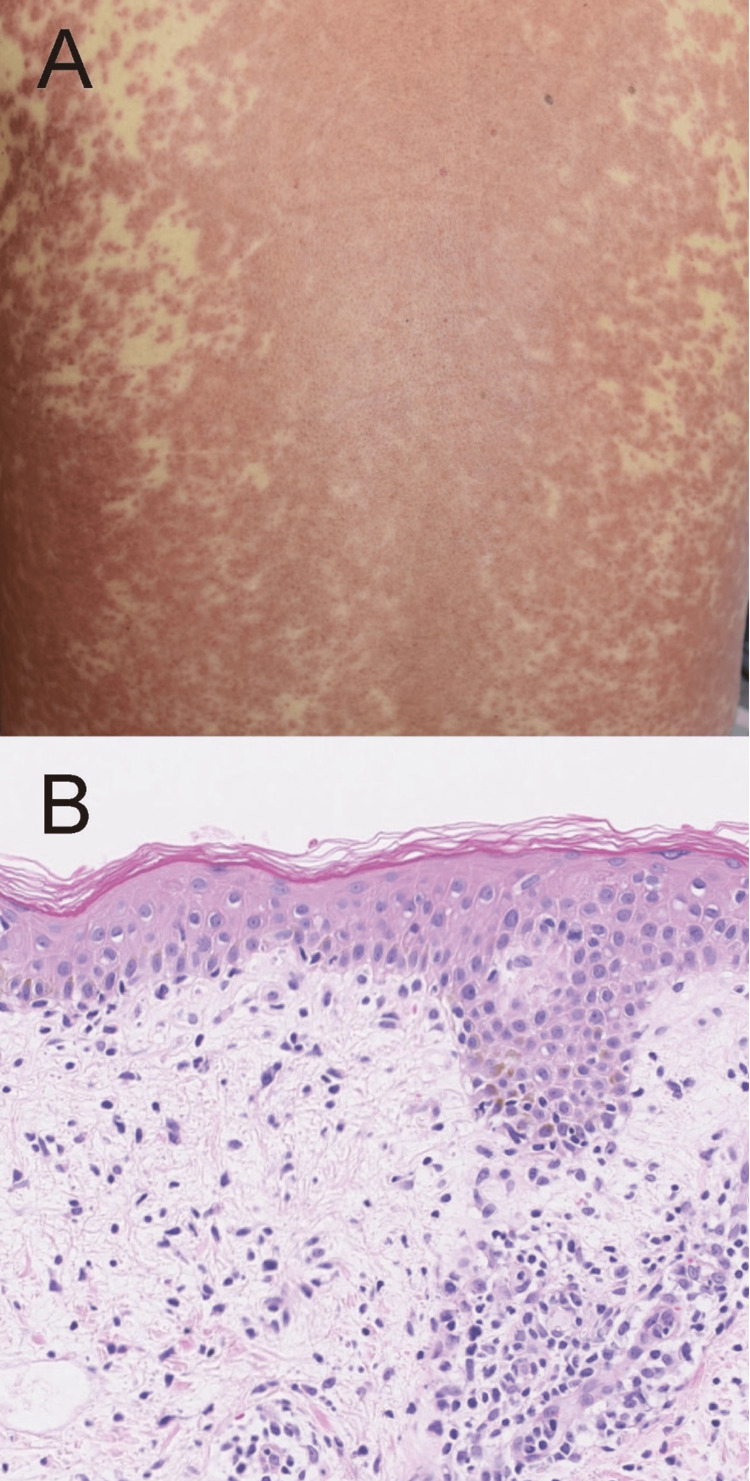
Clinical manifestation and histological examination. (A) Clinical manifestation showing maculopapular skin eruption with follicular accentuation on his whole trunk. (B) A histological examination revealed eczema-like interface dermatitis with infiltrated lymphocytes in the dermis.

A 72-year-old male had allopurinol for his gout and recognized erythematous eruption on his trunk and extremities 28 days after allopurinol administration. He received 300 mg of allopurinol orally every day. He also recognized edema in his extremities 36 days after the treatment. He was also administered anti-hypertensive agents (amlodipine) several years ago. He was referred to our department for evaluation of his skin eruption. The body temperature was 39.3°C, the pulse rate was 111 bpm, and the blood pressure was 119/73 mmHg. Physical examination showed infiltrated erythematous plaques and papules located on his trunk and extremities (Figure 1A). There was no mucosal lesion. A skin biopsy taken from his erythematous eruption revealed a lymphocyte infiltration in the dermis without dyskeratotic keratinocytes in the epidermis (Figure 1B). The biochemical profiles revealed a below-normal serum albumin level at 3.0 g/dL (normal range 4.1-5.1 g/dL). Additionally, all enzyme levels are elevated: alkaline phosphatase (ALP) is at 1,465 U/L (normal range 106-322 U/L), aspartate aminotransferase (AST) is significantly increased at 616 U/L (normal range 13-30 U/L), alanine aminotransferase (ALT) is elevated at 776 U/L (normal range 10-42 U/L), and gamma-glutamyl transpeptidase (g-GTP) surpasses the normal range at 278 U/L (normal range 13-64 U/L). White blood cell count increased at 19,600/μL with elevation of eosinophils (11.0%) and atypical lymphocytes (9.0%). His renal dysfunction gradually developed showing an elevation of serum creatine (2.64 mg/dL). The previous data for creatinine was 1.34 mg/dL. From the clinical course and laboratory examination, we speculated his skin eruption as DIHS possibly because of allopurinol. Therefore, 40 mg oral prednisolone was administered for his skin eruption under the discontinuation of allopurinol. The creatinine levels increased (up to a maximum of 5.43 mg/dL) over a few days because of oliguria and rising pleural effusion. Therefore, hemodialysis was administered twice a week. Hemodialysis was ceased 11 days after hemodialysis administration. However, his skin eruption and renal dysfunction were improved by the treatment. After four weeks, the human herpesvirus (HHV)-6-IgG titer was increased from 1:10 to 1:80. Allopurinol showed a positive reaction in the lymphocyte stimulation test. A Lymphocyte stimulation test using the metabolite of allopurinol, oxypurinol, was not performed, although oxypurinol is a metabolite of allopurinol, and it is believed to be one of the major causative triggers for drug eruption following allopurinol administration [9]. His skin eruption was not re-evoked by the readministration of amlodipine.

## Discussion

Although DIHS is sometimes complicated by acute renal failure, hemodialysis administration for acute renal failure following DIHS is sometimes observed. This is very important information. A previous study showed that 14.8% of cases experienced an increased serum creatinine, and 7.4% of cases required short-term supportive hemodialysis in DIHS patients [8]. Because the detailed clinical characteristics of these cases, especially the prognosis, remained unclear, we reviewed the cases in English or Japanese languages based on previously published case report literature [6-8,10] (Table 1). There have been six reported cases including our case. The male:female ratio is 2:1, and the mean age is 57.8 years. The causative agents are not specific, and a wide variety of drugs become the causative agent to cause acute renal failure with hemodialysis administration following DIHS. The average interval of the initiation of drug intake and the appearance of drug eruption and acute renal failure are 23.0 days and 46.3 days, respectively. Therefore, renal dysfunction was recognized three weeks after the appearance of following skin eruptions on average. All cases well responded to hemodialysis, and they stopped using hemodialysis shortly, suggesting a possible favorable clinical behavior in these cases.

**Table 1:** Review of the cases of hemodialysis administration for acute renal failure following DIHS. ND: not described

Author	Sex	Age	Causative agent	Interval period from causative agent administration		Outcome of renal failure
				Drug eruption	Acute renal failure	
Higuchi et al. [6]	Male	53	Diaphenylsulfone	28 days	47 days	Hemodialysis off
Fujita et al. [7]	Male	29	Zonisamide	44 days	52 days	Hemodialysis off
Augusto et al. [10]	Female	77	Sulphasalazine	28 days	50 days	Hemodialysis off
Ang et al. [8]	Male	32	Ciprofloxacin or metronidazole	3 days	ND	Hemodialysis off
	Female	84	Metformin	7 days	ND	Hemodialysis off
Our case	Male	72	Allopurinol	28 days	36 days	Hemodialysis off

The pathogenesis of acute renal failure following DIHS remains unclear. Interestingly, it has also been reported that the lymphocyte stimulation test is also helpful for finding the causative agent in drug-induced acute renal failure [11]. Furthermore, allopurinol excretes uric acid to renal tubules. These uric acids exacerbate innate immune response-related inflammation through NALP3 inflammasome activation in renal tubules [12].

## Conclusions

Taken together, the combinations of allergic and innate immune inflammations might contribute to the pathogenesis of acute renal failure following allopurinol-induced DIHS. No specific causative agent has been identified in our literature review, and the clinician should keep in mind the risk of severe acute renal failure who might need hemodialysis during the clinical course. As the limited number of cases need hemodialysis in acute renal failure in DIHS cases, further detailed mechanisms or characteristics of acute renal failure in patients with DIHS need further investigation.
